# Treatment of Refractory Vomiting With Traditional Chinese Veterinary Medicine in Two Dogs and a Cat: A Cases Series

**DOI:** 10.1002/vms3.71297

**Published:** 2026-10-30

**Authors:** Xinhui Wang, Letong Yang, Wuren Ma

**Affiliations:** ^1^ College of Veterinary Medicine and Institute of Traditional Chinese Veterinary Medicine Northwest A&F University Xianyang Shaanxi People's Republic of China; ^2^ Xi'an Veterinary Teaching Hospital Northwest A&F University Xi'an Shaanxi People's Republic of China

**Keywords:** refractory vomiting, stomach *Qi* rebellion, syndrome differentiation, Traditional Chinese Veterinary Medicine, two dogs and a cat, Xuanfu Daizhe Decoction

## Abstract

Refractory vomiting in dogs and cats is a common clinical condition in small animal practice, typically characterized by recurrent vomiting, belching and related symptoms, which is a great challenge to veterinarians. According to Traditional Chinese Veterinary Medicine (TCVM), this condition is primarily attributed to dysfunction of the spleen and stomach in transformation and transportation, leading to the upward rebellion of stomach *Qi*. Derived from the *Shang Han Lun* (Treatise on Cold Damage), Xuanfu Daizhe Decoction functions to direct rebellious *Qi* downward, resolve phlegm, tonify *Qi* and harmonize the stomach. It is mainly indicated for conditions resulting from stomach *Qi* counter flow. In this report, three cases of refractory vomiting in two dogs and a cat diagnosed as stomach *Qi* rebellion according to TCVM were treated with modified *Xuanfu Daizhe* Decoction based on syndrome differentiation. The results showed that all three cases achieved favourable therapeutic outcomes. These findings suggest that, for refractory vomiting caused by stomach *Qi* rebellion in dogs and cats, individualized treatment with modified Xuanfu Daizhe Decoction based on specific clinical presentations can achieve stable and reliable efficacy.

## Graphical abstract

Three cases (two dogs and one cat) with refractory vomiting due to Stomach‐Qi rebellion received syndrome differentiated modified Xuanfu Daizhe Decoction. All animals achieved full clinical recovery from vomiting and related symptoms. This classical herbal formula offers an effective TCVM therapeutic option for this condition.

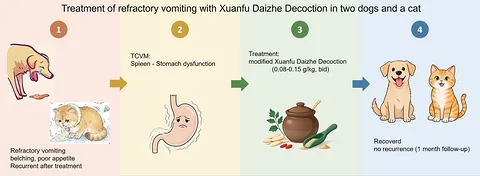

**Abbreviations**
TCVM: Traditional Chinese Veterinary Medicine

## 1 Introduction

Refractory vomiting in dogs and cats is a common and complex gastrointestinal condition in small animal clinical practice, primarily characterized by persistent or recurrent vomiting, belching and decreased appetite, which can severely impair nutritional status and overall health. Conventional Western medical treatments mainly include antiemetics, acid suppression, prokinetic agents and gastric mucosal protectants (Lu and Su 2022). However, in some chronic or recurrent cases, therapeutic efficacy remains unstable, with a high recurrence rate, making it difficult to achieve complete resolution (X. M. Wu et al. 2012)^.^ Therefore, the development of more systematic and effective therapeutic strategies is of considerable clinical importance.

According to Traditional Chinese Veterinary Medicine (TCVM), vomiting is fundamentally attributed to dysfunction of the spleen and stomach, with the key pathogenesis involving failure of the Stomach to maintain its descending function and the consequent upward rebellion of stomach *Qi* (Liu and Xu 2002). The spleen governs transformation and transportation, and its deficiency leads to internal accumulation of dampness and generation of phlegm, while impairment of the stomach's descending function results in anorexia, abdominal distension, belching and vomiting. Accordingly, treatment should focus on harmonizing the stomach and directing rebellious *Qi* downward, combined with syndrome differentiation and guided by the principles of tonifying deficiency, reducing excess, warming the middle burner and clearing heat.

Xuanfu Daizhe Decoction was first recorded in *Shang Han Lun* (Treatise on Cold Damage Diseases) by Zhang Zhongjing (150–219 AD) of the Eastern Han Dynasty. It is indicated for the condition described as: ‘After sweating therapy for cold, or following vomiting or purgation, when the disorder has been resolved but there remains focal distention and hardness below the heart, accompanied by persistent belching’ (Z. J. Zhang 2005). The formula consists of Xuan Fu Hua (Inulae Flos), Ban Xia (Pinelliae Rhizoma), Gan Cao (Glycyrrhizae Radix), Ren Shen (Ginseng Radix), Dai Zhe Shi (Haematitum), Sheng Jiang (Zingiberis Rhizoma Recens) and Da Zao (Jujubae Fructus), and exerts the effects of directing rebellious *Qi* downward, resolving phlegm, tonifying *Qi* and harmonizing the stomach (Yan and Xu 2023; S. S. Li et al. 2022). In clinical settings, it has been widely applied in the management of gastrointestinal disorders such as reflux oesophagitis and gastritis, with well‐documented efficacy (J. J. Zhang and Wu 2015).

Based on TCVM and the evidence‐based application of Xuanfu Daizhe Decoction in human medicine, this study innovatively applies this classical formula to the treatment of refractory vomiting in dogs and a cat. The prescription was modified according to syndrome differentiation to accommodate variations in constitution and clinical manifestations among individual cases. Clinical observations demonstrated favourable therapeutic outcomes in typical cases. The diagnostic and therapeutic processes and clinical experiences are summarized herein, with the aim of providing new insights and references for the management of refractory vomiting in small animal practice.

## 2 Materials and Methods

### 2.1 Case Selection

Animals meeting the following criteria were diagnosed with refractory vomiting and enrolled in the study: (1) presence of chronic or recurrent vomiting and/or persistent belching for at least 2 weeks; (2) poor or incomplete response to previous conventional Western medical treatments; and (3) fulfilment of the diagnostic criteria for ‘stomach *Qi* counter flow’ based on TCVM syndrome differentiation. Cases with severe systemic diseases requiring emergency intervention were excluded. To minimize confounding factors, the present study documented the breed, body size (categorized as small, medium or large), age, habitual diet and season of onset for each case.

### 2.2 Clinical Examination and Diagnosis

All animals underwent routine clinical evaluation, including physical examination, assessment of appetite, observation of defecation and urination and abdominal palpation. Additional diagnostic procedures, such as gastroscopy or contrast radiography, were performed as needed to evaluate gastrointestinal lesions (e.g., gastric ulcers).

TCVM diagnosis was established based on a comprehensive assessment of clinical signs, tongue characteristics and pulse findings, following the principle of syndrome differentiation and treatment. The identified TCVM syndromes in this study included spleen–stomach deficiency cold syndrome and stomach *Yin* deficiency syndrome, both of which are associated with stomach *Qi* counter flow.

### 2.3 Treatment Protocol

All cases were treated with modified Xuanfu Daizhe Decoction based on individual TCVM syndrome differentiation. The classical formula consists of Xuan Fu Hua (Inulae Flos), Ban Xia (Pinelliae Rhizoma), Gan Cao (Glycyrrhizae Radix), Ren Shen (Ginseng Radix), Dai Zhe Shi (Haematitum), Sheng Jiang (Zingiberis Rhizoma Recens) and Da Zao (Jujubae Fructus), with additional herbs incorporated according to clinical presentation. All individual Chinese herbal extract granules were sourced from Beijing Kangrentang Pharmaceutical Co. Ltd. (Beijing, China). To ensure stability, the granules were sealed in light‐resistant containers and stored under cool, dry conditions.

The herbal extracts were prepared, thoroughly mixed, and encapsulated into capsules for oral administration. These procedures were performed by a trained pharmacy technician under standardized protocols. The dosage and formulation were adjusted according to each patient's body weight and TCVM syndrome differentiation. The prescribed dose was 0.08–0.15 g/kg according to body weight, twice daily.

### 2.4 Outcome Assessment

Treatment efficacy was evaluated by the attending veterinarian based on clinical improvement in vomiting frequency, eructation, appetite and overall condition. Resolution of clinical signs and absence of recurrence within 1 month after the end of treatment were defined as criteria for treatment success.

### 2.5 Ethics Statement

This study is a clinical case report of dogs and a cat based on TCVM diagnostic and therapeutic records. No live animal experiments or research‐specific procedures beyond standard clinical care were involved; therefore, review by an Animal Welfare/Ethics Committee was not applicable.

This study enrolled three clinical cases involving two small‐breed dogs and a cat. The clinical data presented in this case report were collected and used with the informed, voluntary consent of the pet owners for academic publication. All identifying information pertaining to the owners and their pets has been fully anonymized.

All TCVM protocols were administered by attending clinicians holding a valid National Licensed Veterinarian Certificate (People's Republic of China), who had completed systematic postgraduate training in TCVM and possessed over 10 years of clinical experience in the discipline. Therapeutic procedures strictly adhered to the clinical practice guidelines issued by the Chinese Association of Traditional Veterinary Medicine (CATVM), complying with relevant national and professional standards.

## 3 Case Reports

Animals included in this study were clinical cases presented to the Veterinary Teaching Hospital of Northwest A&F University (NWAFU), Shaanxi, China, for TCVM treatment of refractory vomiting.

### 3.1 Case One

Diandian (Little Spot), a female intact toy Poodle, 4 years old with 6 kg of body weight. At 2 years of age, she developed abdominal pain accompanied by a prayer posture and was diagnosed with pancreatitis at another animal hospital. Her condition improved after treatment for pancreatitis. Subsequently, similar episodes recurred every winter and were consistently treated as pancreatitis at the same facility.

Prior to presentation at our hospital, the dog exhibited frequent belching and audible sounds of fluid regurgitation through the oesophagus. Appetite, urination and defecation were generally normal, although appetite decreased during episodes, occasionally accompanied by diarrhoea. During physical examination, the patient showed moderate mental status and a thin body condition. The tongue colour was pale with a thick, greasy coating and the pulse was slow and thready. Gastroscopic examination revealed gastric ulcers, along with a large amount of white mucus (Figure 1). Other laboratory tests were unremarkable. Based on TCVM differentiation, the condition was identified as spleen–stomach deficiency cold with stomach *Qi* counter flow.

**FIGURE 1:**
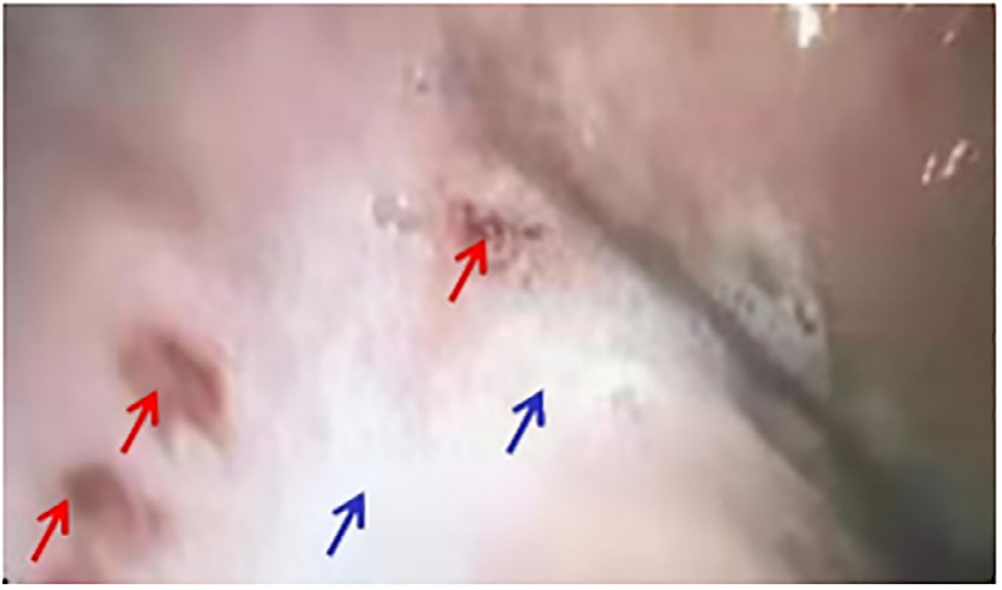
Case 1: Gastroscopy revealed ulcerative lesions (indicated by red arrows) and white gastric juice (indicated by blue arrows) in the gastric mucosa.

The TCVM treatment principle was to warm the spleen and stomach, direct *Qi* downward and stop vomiting. A modified Xuanfu Daizhe Decoction was prescribed: Dai Zhe Shi 60 g (contains 1 g of extract), Xuan Fu Hua 24 g (contains 5 g of extract), Bai Zhu 20 g (contains 6 g of extract), Da Zao 20 g (contains 4 g of extract), Dang Shen 20 g (contains 6 g of extract), Fu Ling 20 g (contains 2 g of extract), Sheng Jiang 12 g (contains 0.5 g of extract), Chen Pi 6 g (contains 1 g of extract). The extract was thoroughly mixed and filled into size No. 1 capsules (0.3 g per capsule) for oral administration. Two capsules were administered each time and twice daily.

*Outcome*. After 2 months of treatment comprising four doses of Chinese herbal powder, the above clinical signs recovered.

### 3.2 Case Two

Lucky, a neutered male toy Poodle, 2 years old with 2.2 kg of body weight, presented with a history of belching lasting 15 days, occasional vomiting and decreased appetite, while urination and defecation remained normal.

On examination, the patient exhibited a moderate mental state and poor appetite, with normal urination and defecation. The tongue colour was pink with a thin coating, and the pulse was thready and rapid. Contrast radiography revealed gastric mucosal injury (Figure 2).

**FIGURE 2:**
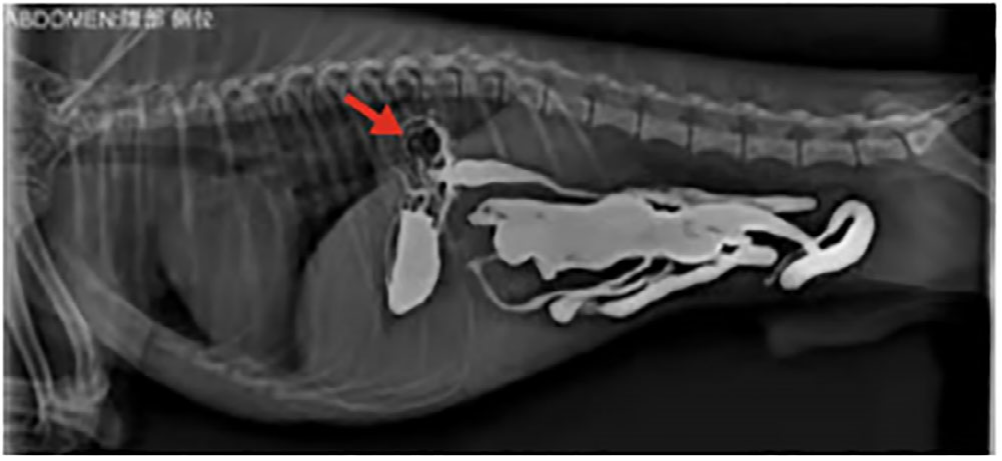
Case 2: Contrast radiography revealed barium retention along the gastric wall, suggesting gastric mucosal injury (indicated by red arrows).

According to TCVM differentiation, the condition was attributed to stomach *Yin* deficiency leading to stomach *Qi* counter flow. The treatment principle was to nourish stomach *Yin*, direct *Qi* downward and stop vomiting. A modified Xuanfu Daizhe Decoction was prescribed: Dai Zhe Shi 30 g (contains 0.5 g of extract), Xuan Fu Hua 12 g (contains 2.5 g of extract), Jiang Ban Xia 12 g (contains 1 g of extract), Zhi Gan Cao 9 g (contains 3 g of extract), Ren Shen 10 g (contains 2.5 g of extract), Sheng Jiang 6 g (contains 0.25 g of extract), Da Zao 10 g (contains 2 g of extract), Zhi Mu 20 g (contains 5 g of extract), Bai Zhu 10 g (contains 3 g of extract), Huang Bai 6 g (contains 0.5 g of extract), Yi Yi Ren 10 g (contains 0.5 g of extract), Fu Ling 10 g (contains 1 g of extract), Tu Si Zi 10 g (contains 0.5 g of extract). The extract was thoroughly mixed and filled into size No. 2 capsules (0.2 g per capsule) for oral administration. One capsule was administered each time and twice daily.

*Outcome*. After 4 weeks of treatment comprising four doses of Chinese herbal powder, the above clinical signs recovered.

### 3.3 Case Three

Heiwa, a male Bengal crossbreed cat, 2.5 years old and 4.8 kg of body weight, was presented on 5 June 2021. Vomiting had begun in May, reaching a maximum frequency of 6–7 episodes per day; at the time of presentation, the frequency had decreased to approximately twice daily. The vomitus consisted of yellow mucus and undigested cat food. The cat showed reduced appetite, decreased faecal output and normal urination.

Clinical examination revealed a fair mental state and mild discomfort upon abdominal palpation. The tongue colour was pale, and the pulse was thready and rapid. Contrast radiography demonstrated gastric mucosal injury (Figure 3).

**FIGURE 3:**
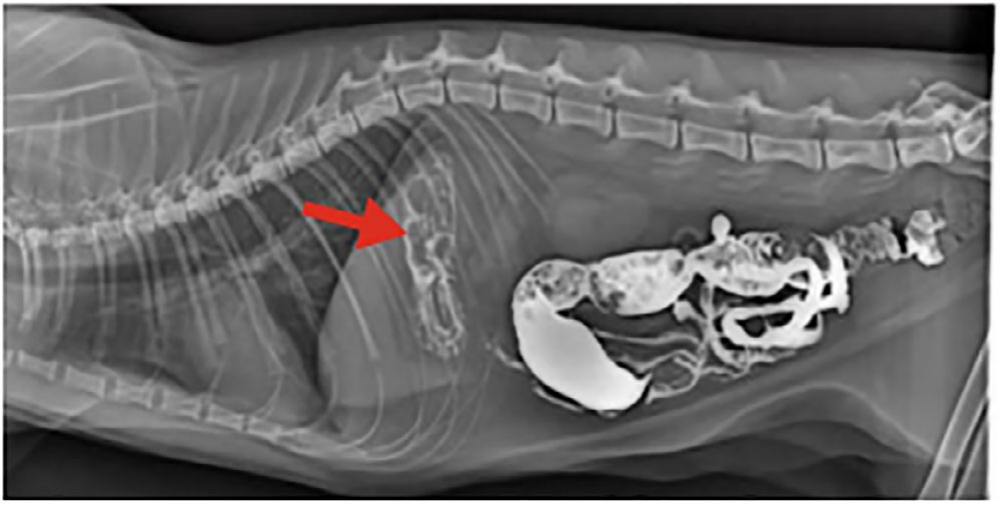
Case 3: Contrast radiography revealed barium retention along the gastric wall, suggesting gastric mucosal injury (indicated by red arrows).

According to TCVM differentiation, the condition was attributed to stomach *Yin* deficiency accompanied by stomach *Qi* counter flow. A modified Xuanfu Daizhe Decoction was prescribed: Duan Dai Zhe Shi 30 g (contains 0.5 g of extract), Xuan Fu Hua 12 g (contains 2.5 g of extract), Jiang Ban Xia 12 g (contains 1 g of extract), Zhi Gan Cao 12 g (contains 4 g of extract), Ren Shen 10 g (contains 2.5 g of extract), Sheng Jiang 15 g (contains 0.625 g of extract), Da Zao 10 g (contains 2 g of extract), Chao Ji Nei Jin 3 g (contains 0.5 g of extract), Jiao Shan Zha 10 g (contains 3 g of extract), Jiao Mai Ya 10 g (contains 0.5 g of extract), Liu Shen Qu 10 g (contains 1 g of extract). The extract was thoroughly mixed and filled into size No. 1 capsules (0.3 g per capsule) for oral administration. Two capsules were administered and twice daily.

*Outcome*. After 1 week of treatment with a single dose of Chinese herbal powder, vomiting resolved, appetite returned to normal and defecation and urination were also normal.

The clinical and demographic characteristics of three cases are summarized in Table 1.

**TABLE 1:** Clinical outcomes of TCVM treatment in two dogs and a cat with refractory vomiting.

Case	Species/breed	Age (years)	Body weight (kg)	typical diet	Season of onset	TCVM pattern	Main clinical signs	Diagnostic findings	Treatment duration	Outcome
1	Dog/toy Poodle	4	6	Commercial diet	Winter	Spleen–stomach deficiency cold	Belching, reflux sounds, decreased appetite, diarrhoea (occasional)	Gastric ulcer (gastroscopy)	2 months (17 January–29 March 2021)	Complete resolution, no recurrence
2	Dog/toy Poodle	2	2.2	Commercial diet	Winter	Stomach Yin deficiency	Belching, occasional vomiting, decreased appetite	Gastric ulcer (contrast radiography)	4 weeks (20 November–23 December 2020)	Complete resolution, no recurrence
3	Cat/Bengal mixed	2.5	4.8	Commercial diet	Spring	Stomach Yin deficiency	Frequent vomiting (up to 6–7/day), decreased appetite, reduced faeces	Gastric ulcer (contrast radiography)	Within 1 week (5–11 June 2021)	Complete resolution, no recurrence

Based on tongue and pulse characteristics, as well as clinical signs, Case 1 was identified as a pattern of spleen–stomach deficiency cold, for which the treatment principle is to warm and strengthen spleen and stomach and dispel cold. Cases 2 and 3 were classified as stomach *Yin* deficiency, for which the treatment principle is to nourish stomach *Yin* while simultaneously directing rebellious *Qi* downward. These findings, in conjunction with imaging examinations, suggest a potential association between gastric mucosal injury and refractory vomiting.

In formula of Xuanfu Daizhe Decoction, Xuan Fu Hua serves as the monarch herb, directing *Qi* downward and resolving phlegm. Dai Zhe Shi, Ban Xia and Sheng Jiang act as minister herbs, suppressing counter flow, stopping vomiting and transforming phlegm and retained fluids. Ren Shen and Da Zao are used as assistant herbs to tonify *Qi* and strengthen spleen and stomach, while Zhi Gan Cao serves as the envoy herb to harmonize the actions of the other ingredients. Through the combined action of these herbs, the formula achieves the effects of directing *Qi* downward, resolving phlegm, tonifying *Qi* and harmonizing the stomach. Consequently, phlegm and retained fluids are eliminated, rebellious *Qi* is subdued, and deficiency of spleen and stomach is restored, thereby relieving epigastric fullness and hardness and alleviating clinical signs such as belching and vomiting.

All three cases showed marked clinical improvement following treatment with modified Xuanfu Daizhe Decoction. Vomiting frequency and belching were significantly reduced in all patients, accompanied by improvements in appetite and general condition. Clinical signs resolved completely in all cases, with no recurrence observed during short‐term follow‐up.

## 4 Discussion

Refractory vomiting in dogs and cats is primarily attributed to dysfunction of the spleen and stomach, with vomiting and belching as the main clinical manifestations. Recurrent episodes and seasonal occurrence suggest an underlying deficiency of *Qi*.

According to the principle of ‘treating different diseases with the same method’ (异病同治) in TCVM, this formula may be applied, with appropriate modifications based on syndrome differentiation, to various conditions caused by spleen–stomach deficiency, internal generation of phlegm–damp, obstruction of *Qi* by phlegm and dysfunction of ascending and descending *Qi* (Y. C. Wu 2006). For patients presenting with belching accompanied by loose stools, a small amount of Rou Gui (Cinnamomi Cortex) may be added (M. Li et al. 2015). In cases of decreased appetite, Liu Shen Qu (Massa Medicata Fermentata), Shan Zha (Crataegi Fructus), Mai Ya (Hordei Fructus Germinatus) and Ji Nei Jin (Galli Gigeriae Endothelium Corneum) may be included to promote digestion, resolve food accumulation and strengthen the spleen. When *Qi* deficiency is pronounced, the proportions of Ren Shen (Ginseng Radix), Da Zao (Jujubae Fructus) and Zhi Gan Cao (Glycyrrhizae Radix) may be increased, and additional herbs such as Dangshen (Codonopsis Radix) and Baizhu (Atractylodis Macrocephalae Rhizoma) may be incorporated, so that sufficient *Qi* can restore the normal ascending and descending functions. For cases characterized by pronounced excess upward rebellion, the dosages of Xuan Fu Hua (Inulae Flos) and Dai Zhe Shi (Haematitum) may be increased to achieve a more rapid therapeutic effect. In cases of severe vomiting caused by impaired transformation and transportation of water and damp in the spleen, the dosages of Ban Xia (Pinellia) and Sheng Jiang (Zingiberis Rhizoma Recens) should be increased, in combination with stir‐fried *Bai Zhu* and Fu Ling (Poria) to strengthen spleen function, promote fluid metabolism and facilitate the elimination of damp. Dosage optimization entailed increasing individual herbs in the base formula by 25%–50%, whereas external herbs serving as adjuvants were introduced at 30%–40% of the principal herb's dosage. Ultimately, this approach aims to restore the balance of *Yin* and *Yang* and re‐establish the proper coordination of the ascending and descending movement of *Qi* within the body. The functions and indications of Xuanfu Daizh*e* Decoction are detailed in Table 2.

**TABLE 2:** The functions and indications of the formulas used in the three cases.

Pin Yin^*^	Latin name	Actions (Xie and Preast 2010)
Dai Zhe Shi	Haematitum	1. Calm the liver and subdue hyperactive *Yang* 2. Exert a heavy, settling action to direct rebellious *Qi* downward 3. Cool the blood and stop bleeding.
Xuan Fu Hua	Inulae Flos	1. Direct *Qi* downward and transform phlegm 2. Redirect rebellious *Qi* to stop vomiting 3. Promote fluid movement and relieve focal distention.
Bai Zhu^a b^	Atractylodis Macrocephalae Rhizoma	1. Tonify the spleen and augment *Qi* 2. Dry dampness and promote urination 3. Stabilize the exterior and stop sweating.
Da Zao	Jujubae Fructus	1. Tonify the middle burner and augment Qi 2. Nourish the blood and calm the mind 3. Harmonize the actions of other herbs.
Dang Shen^a^	Codonopsis Radix	1. Tonify the middle burner and augment *Qi* 2. Strengthen the spleen and benefit the lungs 3. Generate fluids and nourish the blood.
Fu Ling^a b^	Poria	1. Promote urination and leach out dampness 2. Strengthen the spleen and harmonize the middle burner.
Sheng Jiang	Zingiberis Rhizoma Recens	1. Warm the middle burner and dispel cold 2. Redirect rebellious *Qi* to stop vomiting 3. Release the exterior and disperse cold.
Chen Pi^a^	Citri Reticulatae Pericarpium	1. Regulates Qi and strengthens the spleen 2. Dries dampness and transforms phlegm
Jiang Ban Xia	Processed Pinellia	1. Dry dampness and transform phlegm 2. Redirect rebellious *Qi* to stop vomiting and harmonize the stomach.
Zhi Gan Cao	Honey‐fried Glycyrrhizae Radix	1. Tonify the middle burner and augment Qi 2. Relieve spasms and alleviate pain 3. Harmonize the actions of other herbs.
Ren Shen	Ginseng Radix	1. Greatly tonify primordial Qi 2. Strengthen the spleen and benefit the lungs 3. Generate fluids and calm the mind.
Zhi Mu^b^	Anemarrhenae Rhizoma	1. Clear Heat and drain fire 2. Nourish *Yin* and moisten dryness.
Huang Bai^b^	Phellodendri Cortex	1. Lear heat and dry dampness 2. Drain fire and resolve toxicity.
Yi Yi Ren^b^	Coicis Semen	1. Promote urination and leach out dampness 2. Strengthen the spleen and stop diarrhoea 3. Clear heat and discharge pus.
Tu Si Zi^b^	Cuscutae Semen	1. Tonify the kidney and augment Essence 2. Nourish the liver and improve vision 3. Secure essence and stop diarrhoea.
Chao Ji Nei Jin^c^	Processed Galli Gigeriae Endothelium Corneum	1. Promote digestion and resolve food accumulation 2. Strengthen the spleen and enhance transportation 3. Promote urination and dissolve calculi.
Jiao Shan Zha^c^	Charred Crataegi Fructus	1. Promote digestion and resolve food accumulation 2. Invigorate blood and dissipate stasis.
Jiao Mai Ya^c^	Charred Hordei Fructus Germinatus	1. Promote digestion and harmonize the middle burner 2. Strengthen the spleen and stimulate appetite 3. Reduce lactation.
Liu Shen Qu^c^	Massa Medicata Fermentata	1. Promote digestion and resolve food accumulation 2. Strengthen the spleen and harmonize the stomach.

* Pinyin = Hanyu Pinyin romanization of the Chinese name for the herbs. Herbs without superscript letters are those administered to all three cases. In addition, herbs marked with the superscript letter ‘a’ are exclusively used for Case 1, those with the superscript letter ‘b’ for Case 2, and those with the superscript letter ‘c’ for Case 3.

Furthermore, TCVM acupuncture has demonstrated considerable efficacy against vomiting. Stimulation of the Neiguan (PC‐6) acupoint exerts significant anti‐emetic effects and inhibits retrograde peristaltic contractions. In clinical practice, PC‐6 is frequently combined with Zusanli (ST‐36) and Pishu (BL‐21) to manage refractory vomiting secondary to gastrointestinal dysfunction (Tatewaki et al. 2005). Clinical observations suggest that modified Xuanfu Daizhe Decoction yields superior outcomes when integrated with acupuncture therapy.

In clinical medicine, Xuanfu Daizhe Decoction is used for the treatment of gastrointestinal disorders such as reflux oesophagitis and functional dyspepsia, and it can be combined with other formulas according to different aetiologies and path mechanisms to achieve improved therapeutic outcomes (Chen et al. 2023). Y. Y. Zhang et al. (2025) demonstrated that the combination of Liujunzi Decoction and Xuanfu Daizhe Decoction can significantly improve reflux esophagitis caused by spleen stomach deficiency and stomach *Qi* counter flow. Wang Chongwen reported that Xuanfu Daizhe Decoction combined with conventional Western medications such as omeprazole shows good efficacy in the treatment of refractory belching (Wang 2023).

Research has further elucidated the mechanisms underlying the effects of Xuanfu Daizhe Decoction. Studies have shown that modified Xuanfu Daizhe Decoction can regulate the PI3K/AKT signalling pathway, modulate the release of inflammatory cytokines, and influence apoptosis, thereby protecting the integrity of the oesophageal mucosa (Jing et al. 2025). In addition, it can regulate the TFAM‐mtDNA signalling pathway, modulate mitophagy and intervene in precancerous lesions of the oesophagus (Song et al. 2026). These studies indicate that modified Xuanfu Daizhe Decoction exerts multi‐pathway, synergistic regulatory effects, thereby reducing inflammatory responses and promoting ulcer healing.

This study has several limitations that should be considered. First, the age range of the included animals was relatively narrow, which may reduce the applicability of the results to a broader population. Second, treatment outcomes were primarily assessed based on clinical observations, such as vomiting frequency, appetite and general condition, without the use of standardized or objective quantitative measures, which may introduce subjectivity and bias. Future studies with larger and more diverse populations, controlled designs and objective outcome measures are warranted to further validate these findings.

## 5 Conclusion

Xuanfu Daizhe Decoction, a classical formula originating from the *Shanghan Lun* (Treatise on Cold Damage), has the functions of descending counter flow, resolving phlegm, replenishing *Qi* and harmonizing the stomach. This study, based on the observation of three cases of refractory vomiting in two dogs and a cat, verifies the clinical efficacy of the classical formula Xuanfu Daizhe Decoction in treating vomiting and belching caused by impaired spleen and stomach transportation and transformation in small animal medicine.

## Author Contributions

**Xinhui Wang**: data curation, writing – original draft, project administration. **Letong Yang**: data curation, writing – original draft. **Wuren Ma**: conceptualization, funding acquisition, investigation, writing – review and editing.

## Funding

The authors have nothing to report.

## Ethics Statement

All procedures were conducted in accordance with institutional guidelines for animal care and use. Informed consent was obtained from the owners of all animals prior to treatment.

## Conflicts of Interest

The authors declare no conflicts of interest.

## Data Availability

The data that support the findings of this study are available from the corresponding author upon reasonable request.
